# Assessment of the FilmArray Gastrointestinal Pathogen PCR Panel at a Tertiary Cancer Center

**DOI:** 10.1017/ash.2024.218

**Published:** 2024-09-16

**Authors:** Jerin Madhavappallil, Justin Laracy, Mini Kamboj, Judy Yan, Shauna Usiak

**Affiliations:** Memorial Sloan Kettering Cancer Center

## Abstract

**Background:** The FilmArray gastrointestinal (GI) pathogen panel (BioFire Diagnostics, Salt Lake City, UT) is a multiplex PCR assay for syndromic diagnosis of infectious gastroenteritis. This highly sensitive assay has been widely adopted as a preferred testing modality for infectious diarrhea among hospitalized patients. However, in the era of diagnostic stewardship, concerns have been raised that this approach risks unexpected findings of questionable significance. Following an increase in GI pathogen panel testing, the infection control department reviewed results among hospitalized patients at different stages of admission. **Methods:** From October 2022 to May 2023, we retrospectively reviewed all GI pathogen panels sent in a large tertiary cancer hospital. Count of tests ordered and positivity trends were studied by unit and organism among inpatients. We categorized an admission course into early (≤2 inpatient days) and late (≥3 inpatient days) stages and compared results across these stages. Finally, we compared reproducibility of multiple tests sent during a single admission. **Results:** From October 2022 to May 2023, a total of 2,763 tests were sent across the institution with 2,113 tests from inpatient units. Tests were most commonly sent on the Pediatrics and Hematology -Oncology inpatient units and together these units accounted for 60% of tests. These two units also had the highest rate of test positivity and together accounted for 60% of positive tests among hospitalized patients. The most frequently detected organisms were Norovirus (7%) and Enteropathogenic E. coli (3%) (Figure 1). Patients tested in the early stage of hospital admission were more likely to have a positive result for any target (93/509, 18.3%) compared to patients tested in the late stage (202/1604, 12.5%). Patients with a positive test in the early stage of admission were less likely to have a subsequent negative test (3/93, 3%) compared to patients who were positive in late stage of admission (39/202, 19.3% (Figure 2). **Conclusions:** Our findings suggest that the utility of the FilmArray GI PCR panel is highest in the early stages of a patient’s hospital admission. Testing of patients hospitalized ≥3 days is likely to be inappropriate. These findings support implementation of diagnostic stewardship standards on when syndromic testing for potentially infectious diarrhea is appropriate. Figure 1: FilmArray gastrointestinal pathogen PCR panel positivity by organism. Figure 2: FilmArray gastrointestinal pathogen PCR panel positivity by organism comparing early vs late stage of hospital admission.

